# Over-expression, purification, and kinetic analysis of *Mycobacterium tuberculosis* WecA

**DOI:** 10.1080/14756366.2025.2610028

**Published:** 2026-01-08

**Authors:** Yishu Zhao, Haiying Jia, Yan Wang, Shanshan Sha, Dong An, Shufeng Yang, Lei Qian, Yufang Ma, Liming Xu

**Affiliations:** ^a^ Scientific Research Center, The Second Affiliated Hospital of Dalian Medical University, Dalian, China; ^b^ Department of Internal Medicine, The Second Affiliated Hospital of Dalian Medical University, Dalian, China; ^c^ Department of Biochemistry and Molecular Biology, Dalian Medical University, Dalian, China; ^d^ Department of Microbiology, Dalian Medical University, Dalian, China; ^e^ Advanced Institute for Medical Sciences, Dalian Medical University, Dalian, China

**Keywords:** *Mycobacterium tuberculosis*, N-acetylglucosamine-1-phosphate transferase, WecA, membrane protein, tunicamycin

## Abstract

The N-acetylglucosamine-1-phosphate transferase (WecA)is a potential target for developing anti-tuberculosis drugs, due to its critical role in the synthesis of mycobacterial cell wall. The enzymatic study of WecA and the discovery of WecA inhibitors are therefore justified. However, WecA is a membrane protein with 11 transmembrane domains, making it difficult to be obtained, and even more difficult to perform activity studies. In order to gain sufficient WecA protein for activity investigation, the *Escherichia coli* (*E. coli*) Lemo21(DE3) strain was utilised in this study. The expression level of WecA was precisely regulated by T7 lysozyme. Purified WecA was obtained by affinity chromatography and identified by mass spectrometry. The kinetic properties of WecA were determined based on the detection of the product UMP. In addition, tunicamycin proved to be a competitive inhibitor. These results will lay theoretical foundations for the elucidation of WecA catalytic mechanism and the development of WecA inhibitors.

## Introduction

Tuberculosis (TB) is a chronic infectious disease caused by *Mycobacterium tuberculosis* (Mtb). In 2024, there were an estimated 10.7 million new cases of TB and 1.23 million deaths^1^. Furthermore, the emergence of multidrug-resistant (MDR) TB and extensively drug-resistant (XDR) TB, as well as the HIV-associated TB made the disease exceptionally difficult to be treated^2–4^. Therefore, it is required to explore novel TB drugs based on new potential drug targets.

**Figure 1.**
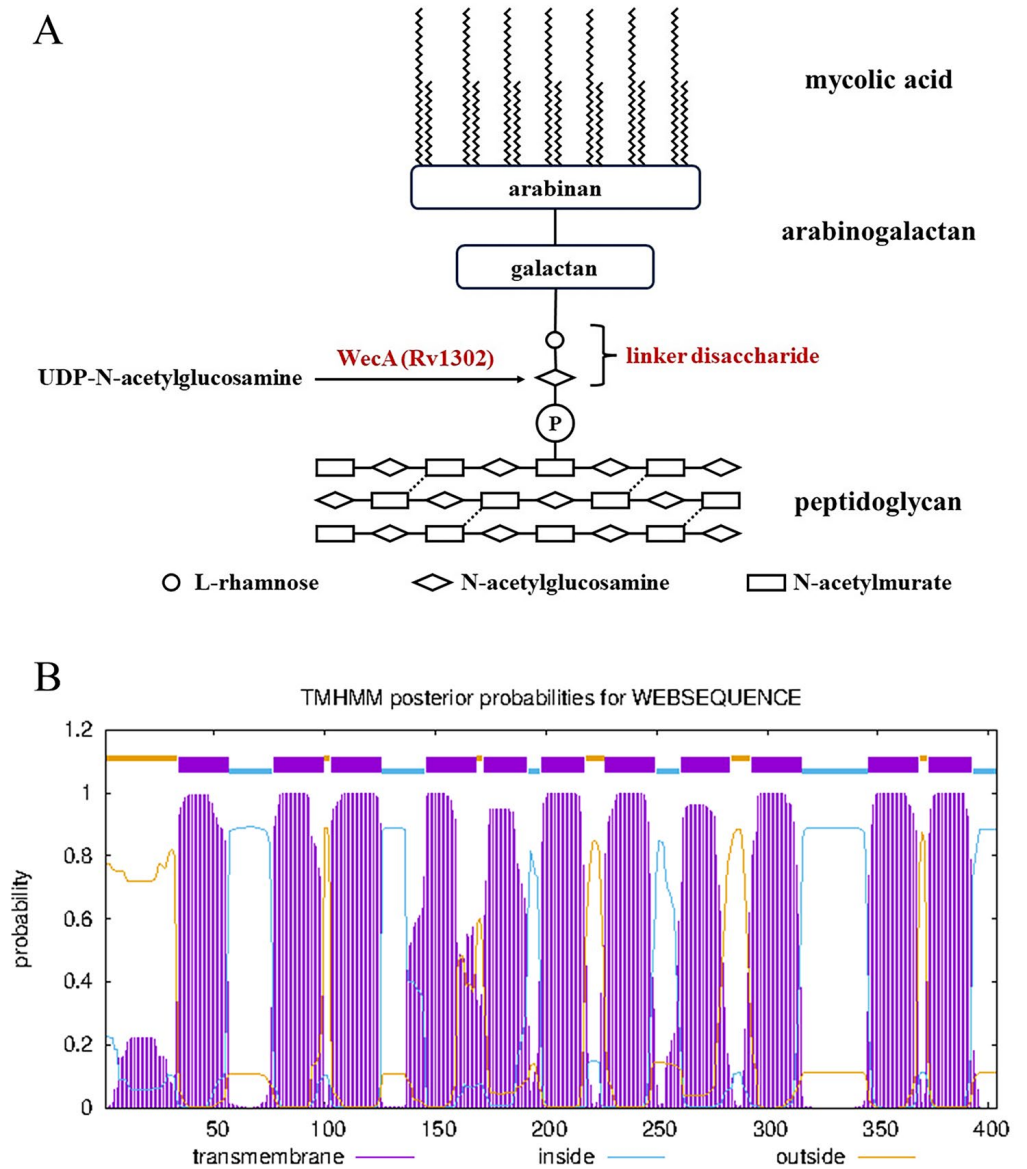
The functional and structural information of WecA. (A) The role of WecA in mycobacterial cell wall core. (B) The transmembrane prediction of WecA by TMHMM tool.

The distinguishing features of the mycobacterial cell wall is a well-recognised library of anti-TB drug targets^5^^,^^6^. The core structure of the mycobacterial cell wall is mainly composed of peptidoglycan (PG), arabinogalactan (AG), and mycolic acids^7^. A disaccharide linker, l-rhamnose-d-N-acetylglucosamine, which covalently attaches AG to PG (Figure 1(A)), is essential for the integrity of the cell wall structure and mycobacterial survival^8^. N-acetylglucosamine-1-phosphate transferase (WecA) catalyses the first step in the synthesis of the linker disaccharide^5^. In the reaction catalysed by WecA, the GlcNAc-1-P group is transferred from UDP-GlcNAc to decaprenyl phosphate (C_50_-P), yielding a glycolipid C_50_-P-P-GlcNAc^8^. Rv1302, the coding gene of WecA was proven to be essential for mycobacterial growth^9^^,^^10^. Therefore, WecA is a potential target for the development of anti-TB drugs. Furthermore, WecA was predicted to be a membrane protein with 11 transmembrane domains (Figure 1(B)). Based on the advantage that drugs targeting membrane proteins do not require cell permeability, WecA furthermore becomes an ideal anti-TB target.

In our previous study, we found that down-regulation of WecA enhanced the sensitivity of mycobacteria against rifampin, indicating that combining treatment with rifampin and WecA inhibitors would enhance the inhibitory effect on TB^11^. Thus, the investigation of the enzymatic properties of WecA and the discovery of effective inhibitors for WecA are well justified. Once inhibitors against WecA are found, the type of inhibition will be determined according to the kinetic parameters of WecA.

The key scientific problems to be solved for WecA enzymatic studies are the acquisition of WecA and the establishment of a high-throughput assay for WecA enzymatic activity. To gain sufficient WecA protein for activity analysis, the *Escherichia coli* (*E. coli*) Lemo21(DE3) strain was utilised in this study. The expression level of target proteins was precisely regulated by T7 lysozyme (LysY) which was induced by rhamnose^12^. In addition, a WecA activity assay was established based on the detection of UMP, the product of the WecA-catalysed reaction. The determination of WecA kinetic properties and investigations on the effect of tunicamycin on WecA enzymatic activity in this study will lay theoretical foundations for the elucidation of WecA catalytic mechanism and the development of the WecA inhibitors.

## Materials and methods

### Plasmids, bacterial strains, and growth conditions

The bacterial strains and plasmids used in this study are listed in Table 1. *E. coli* NovaBlue and Lemo21(DE3) strains were grown in Luria–Bertani (LB) medium. Antibiotic supplements were used as follows: ampicillin (Amp), 100 μg/ml; kanamycin (Kan), 50 μg/ml; chloromycetin (Chl), 34 μg/ml.

**Table 1.** Bacterial strains and plasmids used in this study.

	Description	Source
Strains		
*E. coli* NovaBlue	Used for cloning and propagation of plasmids	Novagen (Cambridge, MA)
*E. coli* Lemo21(DE3)	Used for expressing membrane proteins	NEB
Plasmids		
pJET1.2	Used for cloning PCR products with blunt end	Thermo Scientific (Waltham, MA) (Cat. No. K1231)
pET29b	Used for expressing the WecA and mutation proteins	Novagen (Cambridge, MA) (Cat. No. 69259-3)
pJET-Mtb *wecA*	Mtb *wecA* gene was cloned into the *Eco*RⅤ sites of pJET1.2	This work
pJET-*TEVgfp*	Green fluorescent protein (*gfp*) combined with *Tobacco etch virus* (TEV) protease site were cloned into the *Eco*RⅤ sites of pJET 1.2	This work
pET29b-Mtb *wecA*	Mtb *wecA* gene was cloned into the *Nde*I and *Eco*RI sites of pET29b	This work
pET29b-Mtb *wecA-TEVgfp*	*TEVgfp* gene in pJET was cloned into the *Eco*RI sites of pET29b-Mtb *wecA*	This work

### Purification of WecA protein

#### Construction of pET29b-Mtb wecA expression vector

The *wecA* gene was amplified from Mtb H37Rv genomic DNA by using the primers P1 (GAG*CATATG*CAGTACGGTCTCGAGGTGTC, italicised was *Nde*I site) and P2 (AG*GAATTC*TCCAGGTCCGGGTCGTAGTAG, italicised was *Eco*RI site). The PCR product (1220 bp) was ligated to the pJET1.2/blunt vector, resulting in cloning plasmid pJET-Mtb *wecA*. After resequencing the Mtb *wecA* gene, it was ligated into the *Nde*I and *Eco*RI sites of pET29b vector, yielding the expression plasmid pET29b-Mtb *wecA*. To achieve the detection and purification of WecA protein, the C-terminus of WecA was fused with polyhistidine tag (His-tag) in pET29b vector.

#### Over-expression and purification of WecA membrane protein

The pET29b-Mtb *wecA* plasmid was transformed into the *E. coli* Lemo21(DE3) strain. The recombinant strain was grown in 1000 ml LB medium containing Kan and Chl. Rhamnose (Aladdin, Shanghai, China, Cat. No. 6014-42-2) as an inducer was added at the beginning of the bacterial culture, at a concentration of 0.1 mM which proved to be an optimal concentration for the expression of WecA membrane protein in our previous studies. After a 3 h-incubation at 37 °C, 0.4 mM isopropyl-β-d-thiogalactoside (IPTG, TaKaRa, Kusatsu, Japan, Cat. No. 9030) was added for an additional 16 h-incubation at 30 °C. The cells were harvested by centrifugation (4000 × *g* for 20 min at 4 °C) and resuspended in 60 ml lysis buffer (20 mM Tris–HCl, pH 8.0, 100 mM NaCl, 5% glycerol, 0.1 mM EDTA) with protease inhibitors cocktail (APExBIO, Houston, TX, Cat. No. K100811133C770) and then lysed by sonication (Q125 Sonicator, Qsonica, Newtown, CT). The lysate was centrifuged (8000 × *g* for 30 min at 4 °C) and the supernatant was collected. For the enrichment of membrane protein, the supernatant fraction was ultra-centrifuged (150 000 × *g* for 1.5 h at 4 °C) and the pellet (membrane fraction) was resuspended in 8 ml lysis buffer with 1% (w/v) N-dodecyl-β-d-maltoside (DDM, APExBIO, Houston, TX, Cat. No. C4421) and incubated for 2 h at 4 °C. To further separate the membrane protein, the suspension was ultra-centrifuged again (150 000 × *g* for 30 min at 4 °C) and the supernatant (membrane protein) was applied to 1 ml column volume of Ni-NTA agarose (Qiagen, Hilden, Germany, Cat. No. 30210)^13^. The column was washed by 10 ml washing buffer (20 mM Tris–HCl, pH 8.0, 500 mM NaCl, 20% glycerol, 30 mM imidazole) with 1% DDM and the WecA protein was eluted by 5 ml elution buffer (20 mM Tris–HCl, pH 8.0, 500 mM NaCl, 20% glycerol, 200 mM imidazole) with 1% DDM. The eluate was subjected to an Amicon Ultra-15 Centrifugal Filter (10 kDa MWCO, Millipore, Burlington, MA, Cat. No. UFC8010) to remove small molecules. The purified protein was treated with denaturing and reducing sample loading buffer (EpiZyme, Cambridge, MA, Cat. No. LT101S), followed by separation using Laemmli-type SDS-PAGE (acrylamide at 12% and 5% concentrations in separating gel and stacking gel, respectively) and staining with Coomassie blue R-250 (Solarbio, Beijing, China, Cat. No. C8430)^14^. The band of purified protein in gel was excised and further identified by mass spectrometry (Orbitrap Exploris 480 matched with FAIMS, with ion source of Nanospray) and analysed by using database 1075564acterium + tu–.fasta (Novogene, Beijing, China). For western blot detection, the proteins on the gel were transferred to a nitrocellulose membrane (Pall, Port Washington, NY, Cat. No. 66485) and incubated with mouse derived monoclonal anti-polyhistidine antibody (ABclonal, Woburn, MA, Cat. No. AE003) followed by IRDye 680RD goat anti-mouse secondary antibody (Li-CoR, Lincoln, NE, Cat. No. 926-68070). The protein bands were visualised through 700 nm wavelength channel of the Odyssey CLx imaging system (Li-CoR, Lincoln, NE).

### Assays of WecA activity

The enzymatic activity of WecA was determined by measuring the generation of the product C_50_-P-P-GlcNAc through thin layer chromatography (TLC). The reaction mixture (50 μl), which contained 50 mM Tris–HCl (pH 7.4), 10 mM MgCl_2_, 3 mM UDP-GlcNAc (Promega, Madison, WI, Cat. No. V7071), 0.4 mg/ml C_50_-P (Larodan, Solna, Sweden, Cat. No. 62-1050-4) and 10 µg purified WecA protein, was incubated at 37 °C for 30 min. The reaction solution without the WecA enzyme was used as negative control. Subsequently, 1 ml of chloroform/methanol (2:1) and 250 µl of NH_3_·H_2_O were added to the mixture, and the organic phase was collected after centrifugation at 10 000 × *g* for 10 min. After samples were dried through vacuum centrifugal concentration (Concentrator Plus, Eppendorf, Hamburg, Germany), they were dissolved in 50 µl of chloroform, and 15 µl was loaded on a TLC plate (Silica gel 60 F254, Merck, Rahway, NJ, Cat. No. 1.05554). As a standard, 1 µl C_50_-P was spotted on the TLC plate. The samples were separated into a mobile phase solution (chloroform/methanol/ddH_2_O/1 M ammonium acetate/15 M ammonia hydroxide, 180:140:23:9:9) followed by staining with 10% phosphomolybdic acid (a lipid chromogenic agent) after drying^15^.

### Kinetic studies of WecA enzyme

The kinetic analysis of the WecA enzyme depended on the establishment of a high-throughput assay for the activity detection. In this study, we used the UMP-Glo glycosyltransferase assay kit (Promega, Madison, WI, Cat. No. VA1131), which is a bioluminescent assay based on the detection of UMP, the product of WecA enzymatic reaction. The method converted the UMP into ATP and produced detectable photons in the luciferase reaction. The reactions mentioned above were carried out in a white opaque 384-well plate (Labselect, Hefei, China, Cat. No. 11617). After the enzymatic reactions, an equal volume of UMP detection reagent (containing UMP-Glo enzyme) was added and incubated for 1 h at room temperature according to the manual of the kit. The relative light units (RLUs) value was recorded by the luminescence detection module of a multi-mode microplate reader (SpectraMax i3, MD, San Jose, CA). UMP was used as a reference standard for quantitative analysis. One activity unit of N-actylglucosamine-1-phosphate transferase was defined as the amount of UMP produced per minute.

The kinetic properties of the WecA enzyme were determined by using the UMP-Glo glycosyltransferase assay mentioned above. The initial velocity was determined by performing enzymatic reactions at different concentrations of purified enzyme (2, 5, 10, and 20 μg/ml) and at different incubation times (5, 10, 15, 20, and 25 min). In the range of initial velocity, the optimal temperature, optimal pH, the effect of metal ions such as Mg^2+^ and Ca^2+^ was measured. The optimal pH was measured by performing the enzymatic reaction in 50 mM MES–acetate–Tris (MAT) buffer at a different pH (4.0, 5.0, 6.0, 7.0, 8.0, and 9.0), respectively. The optimal incubation temperature was determined by performing the enzymatic reaction at 16 °C, 30 °C, 37 °C, 42 °C, 50 °C, and 60 °C, respectively. The optimal concentration of Mg^2+^ or Ca^2+^ was measured by adding MgCl_2_ or CaCl_2_ at final concentrations of 0, 2.5, 5, 10, and 20 mM in the enzymatic reaction, respectively. At the optimal conditions, kinetic parameters of WecA enzyme were determined. The *K*_m_ and *V*_max_ values of WecA enzyme for UDP-GlcNAc were measured by performing the enzymatic reaction with various concentrations of UDP-GlcNAc (0.01, 0.02, 0.05, 0.075, and 0.1 mM) and C_50_-P at a saturating concentration of 0.1 mg/ml. The *K*_m_ and *V*_max_ values of WecA enzyme for C_50_-P were measured by performing the enzymatic reaction with various concentrations of C_50_-P (0.01, 0.02, 0.05, 0.075, and 0.1 mg/ml) and UDP-GlcNAc at a saturating concentration of 0.1 mM. The catalytic constant *k*_cat_ was calculated through dividing the *V*_max_ value by the concentration of WecA enzyme (0.045 μM, which was equivalent to 2 μg/ml in enzymatic reactions). In addition, the catalytic efficiency of WecA was measured by the ratio *k*_cat_/*K*_m_.

### The inhibitory effect of tunicamycin on WecA

Tunicamycin is the natural inhibitor of bacterial WecA, which mimics the substrate UDP-GlcNAc through similar molecular structure^16^. To test the inhibitory effect of tunicamycin on WecA, the apparent *K*_m_ and *V*_max_ values of WecA for UDP-GlcNAc in the presence of tunicamycin (Selleck, Houston, TX, Cat. No. S7894) at concentrations of 10 μM, 20 μM, and 50 μM were measured by performing the enzymatic reaction with various concentrations of UDP-GlcNAc and C_50_-P at a saturating concentration as described above.

### Statistical analysis

The data of WecA enzymatic activity were recorded and calculated by using the MS Excel software (Redmond, WA). The mean values and standard deviations from triplicate experiments were used for curve fitting which completed by using GraphPad Prism software (San Diego, CA).

## Results

### The purified WecA protein was obtained

**Figure 2.**
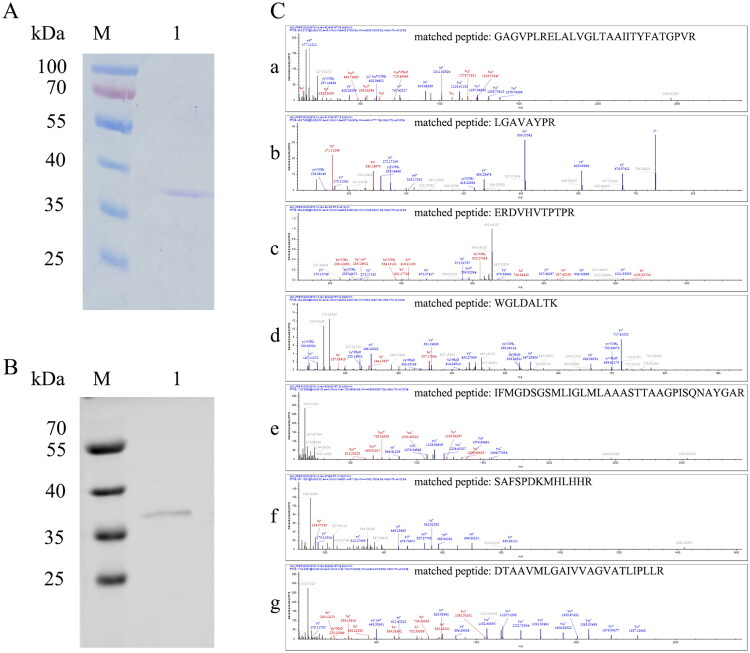
Detection of purified WecA protein. (A) SDS-PAGE (Laemmli-type) detection of purified WecA protein under reducing condition; (B) Western blot detection of purified WecA protein through monoclonal anti-polyhistine antibody. (M) PageRuler™ Prestained Protein Ladder (Thermo Scientific, Waltham, MA); lane 1: the purified WecA protein. Notably, the apparent molecular weight of the purified protein was not consistent with the molecular weight of WecA (45 kDa). It is common phenomenon that membrane proteins observed lower molecular weights in SDS-PAGE. (C) Identification of purified WecA protein by mass spectrometry. (a–g) Spectrograms of matched peptides.

The WecA protein was expressed in *E. coli* Lemo21(DE3) under the induction of 0.1 mM rhamnose and 0.4 mM IPTG. The protein was purified by Ni-NTA affinity chromatography. The results of SDS-PAGE (Figure 2(A)) and Western blot (Figure 2(B)) detection showed that the purified protein was obtained, although the apparent molecular weight of the purified protein was not consistent with the molecular weight of WecA (45 kDa). It is a common phenomenon that membrane proteins are observed at lower molecular weights in SDS-PAGE^17^. The band of purified protein in the gel was further identified by mass spectrometry (Figure 2(C)). The result showed that the purified protein was the WecA protein (Rv1302).

### The purified WecA had the N-acetylglucosamine-1-phosphate transferase activity

**Figure 3.**
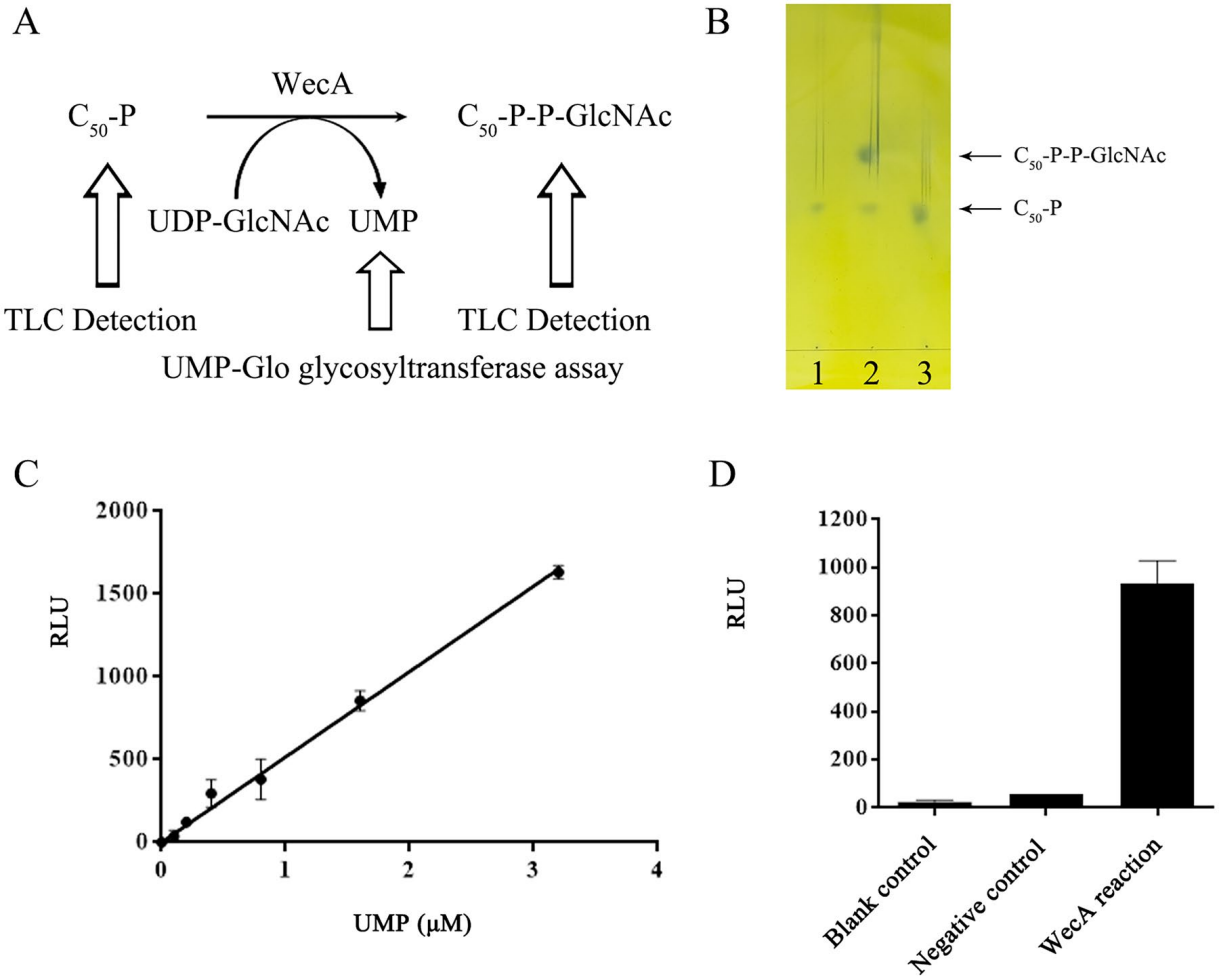
The activity assay of purified WecA protein. (A) The scheme of the WecA activity assay. (B) TLC assay of WecA activity. Lane 1: negative control, the reaction lacking WecA enzyme; lane 2: the enzymatic reaction with WecA; lane 3: C_50_-P standard. (C) The UMP standard curve of UMP-Glo glycosyltransferase assay. The values plotted were the mean value from duplicate experiments. (D) The UMP-Glo glycosyltransferase assay of WecA activity. The values plotted were the mean values and standard deviations from triplicate experiments.

The activity of WecA was determined by TLC analysis through the detection of the product C_50_-P-P-GlcNAc (Figure 3(A)). The results showed that the formation of C_50_-P-P-GlcNAc was observed (Figure 3(B)), indicating that the purified WecA protein had the N-actylglucosamine-1-phosphate transferase activity.

In order to establish a high-throughput assay for the kinetic analysis of WecA, a bioluminescent assay based on the detection of UMP, was used in this study (Figure 3(A)). The results showed that the RLUs of the reactions were dose dependent on UMP (Figure 3(C)). Moreover, compared with negative control group, the amount of UMP in the WecA enzymatic reaction group was obviously increased (Figure 3(D)). The results further demonstrated that the purified WecA protein had the activity of N-actylglucosamine-1-phosphate transferase. Moreover, the bioluminescent assay was suitable for WecA kinetic analysis and high-throughput screening of WecA inhibitors in future studies.

### The kinetic properties of WecA enzyme were measured

**Figure 4.**
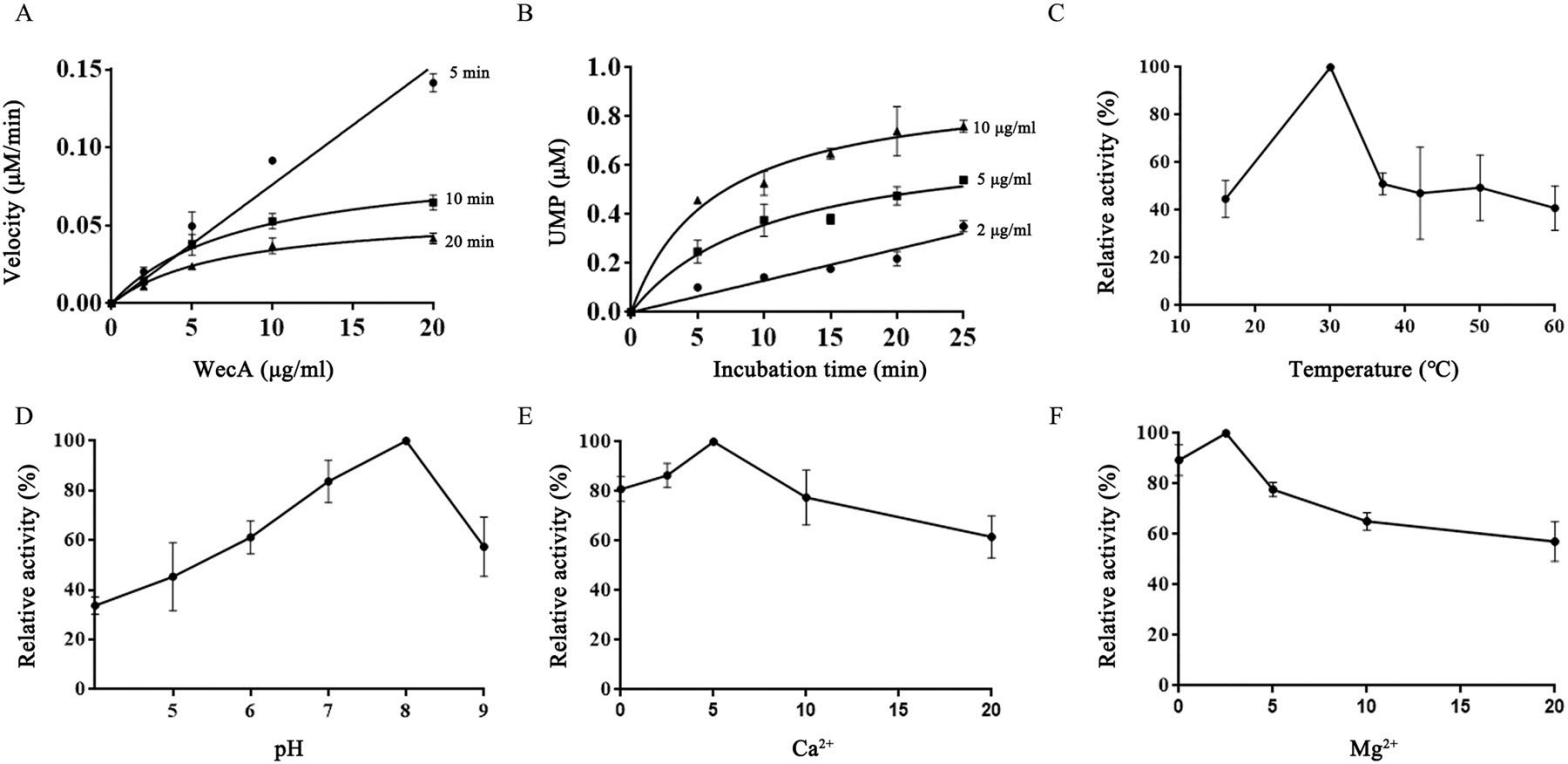
The optimal conditions of WecA enzymatic reactions. (A) Enzyme concentration curves of WecA activity; (B) time course curves of WecA activity; (C) the effect of temperature on WecA activity; (D) the effect of pH on WecA activity; (E) the effect of Ca^2+^ on WecA activity; (F) the effect of Mg^2+^ on WecA activity. The values plotted were the mean values and standard deviations from triplicate experiments.

**Figure 5.**
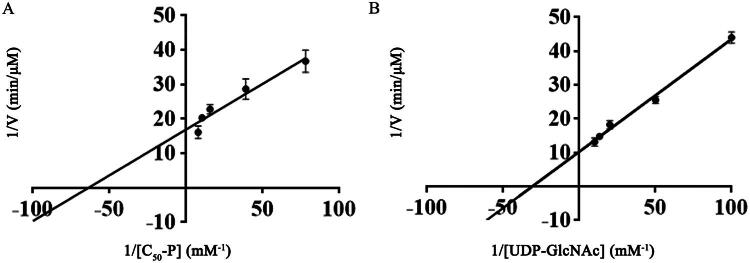
Determination of *K*_m_ and *V*_max_ of WecA from double reciprocal plot. (A) The *K*_m_ and *V*_max_ values of WecA for substrate C_50_-P; (B) the *K*_m_ and *V*_max_ values of WecA for substrate UDP-GlcNAc. The values plotted were the mean values and standard deviations from triplicate experiments.

The UMP-Glo glycosyltransferase assay was utilised to study the kinetic properties of WecA enzyme. The results demonstrated that based on the incubation time and enzyme concentration curves, the time range of initial velocity was limited within 5 min (Figure 4(A)) and 2 μg/ml was the most suitable concentration of WecA enzyme (Figure 4(B)). The WecA enzyme had a relative maximal activity at 30 °C (Figure 4(C)) and pH 8.0 (Figure 4(D)) containing 5 mM Ca^2+^ (Figure 4(E)) and 2.5 mM Mg^2+^ (Figure 4(F)). Under the optimal conditions determined above, the *K*_m_ and *V*_max_ values of WecA enzyme for substrate UDP-GlcNAc and C_50_-P were calculated from double reciprocal plots (Figure 5). The *K*_m_, *V*_max_, *k*_cat_, and *k*_cat_/*K*_m_ values of the WecA enzyme for each substrate are listed in Table 2.

**Table 2.** Kinetic parameters of WecA enzyme.

Substrate	*K*_m_ (μM)	*V*_max_ (μM min^−1^)	*k*_cat_ (s^−1^)	*k*_cat_/*K*_m_ (μM^−1^ s^−1^)
C_50_-P	15.855 ± 2.976	0.059 ± 0.003	0.022 ± 0.001	(1.422 ± 0.212) × 10^−3^
UDP-GlcNAc	32.522 ± 3.308	0.097 ± 0.008	0.036 ± 0.003	(1.114 ± 0.046) × 10^−3^

### Tunicamycin was a competitive inhibitor of WecA enzyme

**Figure 6.**
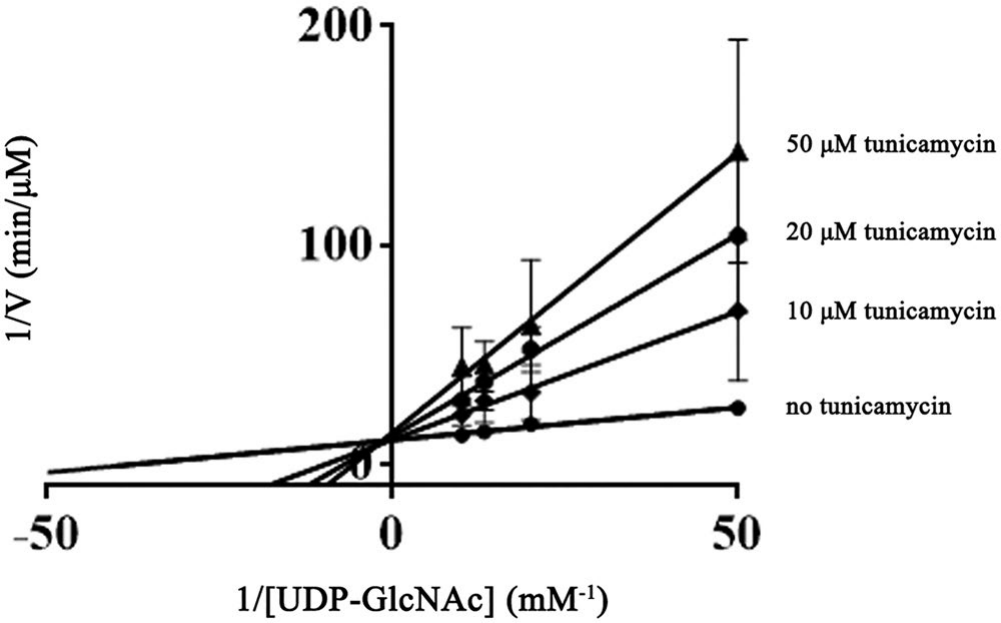
The effect of tunicamycin on WecA activity. The values plotted were the mean values and standard deviations from triplicate experiments.

Tunicamycin at three different concentrations was added to the WecA enzymatic reaction to test its effect on WecA activity. Through the double reciprocal plots, it was revealed that the apparent *K*_m_ of WecA enzyme for UDP-GlcNAc increased along with the increasing of tunicamycin, while there was no significance change in the value of *V*_max_ (Figure 6), indicating that tunicamycin was a competitive inhibitor of WecA enzyme.

## Discussion

Bacterial integral membrane proteins play important roles in cell wall synthesis, environment sensing, antibiotic efflux, nutrient uptake, toxin export, and bacteria–host interaction^18^. In mycobacteria, membrane proteins are also involved in the pathogenicity and susceptibility to host immune recognition. Thus, membrane proteins could be potential targets for the development of new anti-TB drugs^19^. Recently multiple studies on anti-TB targets focused on the structures of membrane proteins (such as Mmpl3, EmbA/B/C family, and AftA) which are known targets or potential targets of drugs^6^^,^^20^^,^^21^. The WecA in this study, which initiates AG biosynthesis, is a membrane protein with 11 predicted transmembrane domains (TMHMM online server and AlphaFold modelling). Due to the essentiality of WecA transformed into the mycobacterial survival^9^^,^^10^, WecA is an ideal target to develop new anti-TB drugs. WecA in *E. coli* has been characterised to catalyse the synthesis of lipopolysaccharides (LPSs)^22^. In the previous studies of our group, Mtb Rv1302 gene was transformed to *E. coli* WecA-defective strain (MV501) constructed by Alexander and Valvano^23^. The synthesis of LPS in *E. coli* MV501 strain was restored upon the complementation with Mtb Rv1302, confirming that Rv1302 was the coding gene of Mtb WecA^10^. Furthermore, we found that down-regulation of WecA enhanced the sensitivity of mycobacteria against rifampin^11^, indicating that the combined treatment with rifampin and WecA inhibitors would enhance the inhibitory effect on TB. These findings motivated the enzymatic study of WecA and the development of WecA inhibitors. However, due to the complex topological structure of WecA, it is hard to over-express in host cells such as *E. coli* and *M. smegmatis*. Furthermore, the extraction and purification of WecA are even more difficult. The studies of mycobacterial WecA enzymes were mostly performed with crude enzymes^24^^,^^25^. It is difficult to ensure the accuracy of the results because of the complexity of the crude enzyme components.

In order to gain sufficient purified WecA protein for enzymatic analysis, multiple approaches including the utilisation of different vectors, host strains and in-fusion tags, as well as *in vitro* (cell-free) expression, were performed in our previous studies. Unfortunately, little WecA protein was obtained. We assumed that the insolubility and the misfolding of membrane proteins were the main reasons for the difficulties experienced with WecA expression and extraction. Therefore, a suitable expression system that enables membrane protein to express in soluble and properly folded form is mandatory. In this study, *E. coli* Lemo21(DE3) strain (NEB, Ipswich, MA), a LysY inducible strain, was used for the expression of WecA. The expression level of WecA was precisely regulated by LysY, which was induced by rhamnose. The Lemo21(DE3) strain proved to be suitable for the expression of membrane proteins of interest^12^. In order to determine the optimal conditions for the expression of WecA, green fluorescent protein (GFP) was used as a reporter in our previous studies. After the detection of GFP expression was monitored by microplate reader, fluorescence microscope and western blot, we determined that 0.1 mM was the optimal concentration of rhamnose for the expression of WecA in the Lemo21(DE3) system (data not shown). Under the optimal conditions, WecA-GFP was expressed in soluble form, but could not be purified by Ni-NTA. We assumed that due to the complex structure of WecA-GFP, the His-tag was not exposed, making it impossible to be purified by Ni-NTA affinity chromatography. Therefore, Lemo21(DE3) carrying pET29b-Mtb *wecA* plasmid was used to express WecA protein directly under the optimal condition determined above. Finally, we obtained sufficient purified WecA protein for enzymatic analysis.

The production of C_50_-P-P-GlcNAc was detected through TLC, indicating that the purified WecA protein had the expected N-actylglucosamine-1-phosphate transferase activity. However, the time-consuming and quantitative unsuitability of the TLC method was not suitable for WecA kinetic analysis in this study, and high-throughput screening of WecA inhibitors in future studies. Therefore, it was necessary to establish a rapid and sensitive assay of WecA activity. The UMP-Glo glycosyltransferase assay kit (Promega, Madison, WI), which is a bioluminescent assay based on the detection of UMP, is suitable for WecA kinetic analysis in this study. By using this assay, the kinetic properties of WecA were determined. The *K*_m_ values of WecA for UDP-GlcNAc and C_50_-P were 32.52 μM and 15.86 μM, respectively. *K*_m_ is a parameter that represents the affinity between the enzyme and the substrate, which means that the larger the *K*_m_, the lower affinity between the enzyme and the substrate. It was found that the affinity of WecA for C_50_-P was higher than that for UDP-GlcNAc. Furthermore, compared with the *K*_m_ values of WecA in other species, such as *T. maritima* (0.62 mM for substrate UDP-GlcNAc and 0.12 mM for substrate C_55_-P) and *E. coli* (0.19 μM for substrate UDP-GlcNAc)^26^^,^^27^, we found that the affinity of Mtb WecA for substrates was higher than that of *T. maritima*, but lower than that of *E. coli*. The catalytic constant *k*_cat_ (*V*_max_/concentration of enzyme) represents the molecular number of substrates converted by each enzyme molecule per second, thus is a parameter that reflects how fast the reaction is. The ratio *k*_cat_/*K*_m_ is a parameter that represents the catalytic efficiency of the enzyme. Therefore, the acquisition of kinetic parameters of WecA in this study will lay a theoretical foundation for the elucidation of WecA catalytic mechanism and the development of WecA inhibitors in future studies, such as caprazamycin and its derivate CPZEN-45^25^.

Tunicamycin, a natural antibiotic which is produced by *Streptomyces lysosuperficus*, was considered as an inhibitor of bacterial WecA^28^. Tunicamycin has been widely used in prokaryotic and eukaryotic studies. However, tunicamycin had not been an anti-TB drug candidate due to its cytotoxic effect through the inhibition of N-glycosylation of eukaryotic proteins^29^. Encouragingly, recent studies found that the selective hydrogenation compounds of tunicamycin (TunR1 and TunR2) retained the antibacterial activity, but had lower mammalian toxicity than tunicamycin^30^^,^^31^. These findings provided the possibility for the modified tunicamycin to become a candidate drug for anti-TB treatment. The results determined in our study confirmed that tunicamycin was a competitive inhibitor of WecA, which was consistent with the observation that the structure of tunicamycin is similar to UDP-GlcNAc, the donor nucleotide sugar. Based on the kinetic properties of WecA determined in this study and the crystal structure as well as the catalytic mechanism of WecA elucidated in future studies, it might be possible to perform new molecular structural modified tunicamycin, making it only effective against mycobacterial WecA, but have no side effects on eukaryotic cells.

In summary, we have established the basis for research in this study that can be used for the studies of WecA protein-based mechanisms and the development of WecA-targeted treatments.

## Data Availability

The data that support the findings of this study are available from the corresponding author (Liming Xu), upon reasonable request.
